# Substrate-Driven Modulation of Nutritional Composition and Bioactive Compound Profile in *Pleurotus pulmonarius* Cultivated on Diversified Agri-Waste

**DOI:** 10.3390/foods15132404

**Published:** 2026-07-07

**Authors:** Monika Kalinowska, Marzena Smolewska, Ewelina Gołębiewska, Aneta Ignaciuk, Grzegorz Świderski, Małgorzata Zawadzka, Ewa Zapora, Maria Saeed, Wala Karar, Lalita Ambigai Sivasamugham, Prakash Balu, Geetha Subramaniam

**Affiliations:** 1 Department of Chemistry, Biology and Biotechnology, Faculty of Civil Engineering and Environmental Science, Institute of Civil Engineering and Energetics, Bialystok University of Technology, Wiejska 45E Street, 15-351 Bialystok, Poland; m.smolewska@pb.edu.pl (M.S.); a.ignaciuk@pb.edu.pl (A.I.);; 2 NatureTECH Centre for Natural Product Research, Bialystok University of Technology, Wiejska 45A Street, 15-351 Bialystok, Poland; 3 Department of Silviculture and Forest Utilization, Faculty of Civil Engineering and Environmental Science, Institute of Forest Sciences, Bialystok University of Technology, Wiejska 45E Street, 15-351 Bialystok, Poland; 4 Faculty of Health and Life Sciences, INTI International University, Persiaran Perdana BBN, Putra Nilai, Nilai 71800, Negeri Sembilan, Malaysialalitaa.sivasamugham@newinti.edu.my (L.A.S.); 5 Department of Biotechnology, Vels Institute of Science, Technology and Advanced Studies (VISTAS), Velan Nagar, P.V. Vaithiyalingam Road, Pallavaram, Chennai 600 177, Tamil Nadu, India; prakazbt@gmail.com

**Keywords:** *Pleurotus pulmonarius*, agri-waste, nutraceutical, food nutrition improvement, agricultural production

## Abstract

*Pleurotus pulmonarius* (Fr.) Quél. is a commercially important edible mushroom recognized for its nutritional and nutraceutical value. However, the influence of alternative agricultural waste substrates on its biochemical composition remains insufficiently characterized. This study investigated the effect of four cultivation substrates, coconut waste (PpC), paddy husk (PpP100), paddy husk supplemented with sawdust (PpP20) and rubberwood sawdust as control (PpS), on the macro- and microelement profile, secondary metabolite composition, and antioxidant activity of *P. pulmonarius* fruiting bodies. Analytical methods included ICP-MS and FAAS for elemental analysis; GC-MS for fatty acid, carbohydrate and phenolic profiling; Kjeldahl method for total protein; FTIR spectroscopy for structural characterization; and four complementary antioxidant assays (DPPH, ABTS, CUPRAC, FRAP). Coconut waste substrate promoted the highest protein accumulation and elevated concentrations of iron, zinc, and specific phenolic acids (vanillic, protocatechuic). Paddy husk-based substrates favored carbohydrate accumulation, particularly trehalose, while sawdust supported the greatest lipid content, dominated by linoleic acid. Potassium was the predominant macroelement across all variants. Antioxidant activity was highest in PpP100 and PpC across all four assays. FTIR confirmed a mushroom-specific polysaccharide, protein, and lipid profile in all samples. The results demonstrate that agricultural waste represents sustainable, value-added alternatives to conventional sawdust, capable of maintaining or enhancing the nutritional and nutraceutical quality of oyster mushrooms to enhance agricultural production.

## 1. Introduction

Mushrooms have always held significant value since ancient times due to their nutritional and medicinal properties [1]. Edible mushrooms are considered functional food because, apart from providing essential nutrients, they contain bioactive compounds that offer extra health benefits. It is estimated that there are 14,000 species of mushrooms in the world, of which 2000 are considered edible and around 200 types of mushrooms are professionally cultivated [2].

One of these species of edible mushrooms that are cultivated commercially is *Pleurotus pulmonarius*, also known as oyster mushroom, which is among the most widely consumed mushrooms [3]. Oyster mushrooms are ranked as the third most widely cultivated mushrooms worldwide, due to their nutritional value and relatively simple production requirements. This mushroom species obtains nutrients through the decomposition of various agricultural by-products [4]. *Pleurotus ostreatus* (Jacq.) P. Kumm. and *Pleurotus pulmonarius* (Fr.) Quél. are saprotrophic basidiomycetes belonging to the family Pleurotaceae (Agaricales, Basidiomycota), widely distributed worldwide and commonly colonizing decaying hardwood, where they play an important role in the biodegradation of lignocellulosic substrates. The nutritional composition of *P. pulmonarius* is particularly noteworthy, as it serves as an excellent source of both macronutrients including proteins, carbohydrates, and dietary fiber, as well as essential micronutrients such as vitamins (particularly B-complex vitamins) and minerals (iron, zinc, potassium, and selenium) [5]. Understanding the macro- and micronutrient profiles of mushrooms cultivated on different substrates is crucial for optimizing their nutritional value, ensuring food security, and meeting dietary requirements of diverse populations, especially in regions where protein malnutrition and micronutrient deficiencies remain prevalent public health concerns.

In Malaysia, mushroom cultivation is traditionally carried out using rubberwood sawdust as the main substrate [6]. However, increasing awareness of forest conservation, which indirectly limits the expansion of rubber plantations, has led to reduced availability of sawdust. Therefore, the use of agricultural residues and other agro-industrial by-products is being increasingly considered as an alternative substrate source. According to a report published in 2021, approximately 1.2 million tons of agricultural waste are generated annually in Malaysia, a significant proportion of which is disposed of in landfills [7]. The accumulation of agricultural waste poses environmental and health concerns. Mushroom cultivation using biotechnological techniques can mitigate these issues by utilizing agricultural waste as a substrate, reducing pollution and health risks associated with improper waste disposal [8].

This study aims to investigate the differences in mycelium growth, and the macro- and micronutrient content of *P. pulmonarius* mushrooms cultivated using various agri-waste materials which are coconut husk and chips, paddy husk supplemented with sawdust in different ratios, and sawdust (from rubber trees) as the control substrate. A comprehensive profile of phenolic compounds, together with a nutritional analysis of both macronutrients (protein, carbohydrate, and fat content) and micronutrients (mineral composition), will provide critical insights into how substrate composition influences the nutritional quality of harvested mushrooms, thereby informing best practices for sustainable cultivation that maximizes both yield and nutritional value. This is in line with the United Nations Sustainable Development Goal 12 which involves responsible consumption and production.

## 2. Materials and Methods

### 2.1. Materials

Methanol for LC-MS, ethanol 99.9% gradient grade (LiChrosolv), hydrogen peroxide (H_2_O_2_) (EMSURE ISO), diethyl ether (>99.8%, ACS reagent), and N,O-bis(trimethylsilyl)trifluoroacetamide with 1% trimethylchlorosilane (BSTFA, derivatization grade 99%) were provided by Supelco. Pyridine anhydrous 99.8%, ethyl acetate HPLC Plus 99.9%, nitric acid 70% (≥99.999% trace metals basis), potassium persulfate (K_2_S_2_O_8_), iron(III) chloride (FeCl_3_·6H_2_O), DPPH (2,2-diphenyl-1-picrylhydrazyl), ABTS (2,2′-azino-bis(3-ethylbenzothiazoline-6-sulfonic acid)), TPTZ (2,4,6-Tris(2-pyridyl)-s-triazine), and acetate buffers (pH 7.0 and pH 3.6) were obtained from Sigma-Aldrich. Sodium sulfate (Na_2_SO_4_) anhydrous pure p.a., boric acid (H_3_BO_3_) pure p.a., sodium hydroxide (NaOH) pure p.a., and ethanol were purchased from Chempur. Indium nitrate (In(NO_3_)_3_) in 2% HNO_3_ and analytical standards of sugars, phenolic acids and fatty acids were purchased from Merck. Multi-element standard (Analityk-170 standard solution VIII in 7% HNO_3_, INORGANIC VENTURES, Analityk-154 standard solution VIII in 5% HNO_3_, INORGANIC VENTURES). Hydrochloric acid (HCl) 36.5% from Baker INSTRA-ANALYZED. Copper(II) chloride (CuCl_2_) was obtained from Alfa Aesar. Diethyl ether ≥99.8% was provided by ACS reagent. Sulfuric acid (H_2_SO_4_) 95% was purchased from ChemSolve. Catalytic mixture KjTabs VTCT were purchased from VelpScientifico. Phenol was provided by Warchem. Neocuproine was purchased from J&K Scientific. Methanol was obtained from POCH.

### 2.2. Cultivation of Pleurotus pulmonarius

#### 2.2.1. Substrate Materials and Preparation

*Pleurotus pulmonarius* spawn, sawdust, calcium carbonate (CaCO_3_), and corn bran were obtained from NasAgro farm in Selangor Malaysia. Coconut husk and chips were sourced from Seremban market, while paddy husk was procured from Brilliant Supplies Sdn. Bhd., Malaysia.

Four substrate formulations were evaluated: paddy husk (100%), paddy husk (20%) supplemented with sawdust (80%), coconut waste (100%), and sawdust (100%) as control. All substrates were prepared following standardized procedures. Sawdust was dried overnight at 60 °C. Coconut waste and paddy husk were dried at 60 °C overnight, then soaked in water for 5–7 h. Paddy husk was ground using a Shimizu grinder for 5 s prior to soaking. Excess moisture from soaked substrates was removed by spinning in cotton cloth bags using a washing machine for 3 min.

#### 2.2.2. Spawn Bag Preparation

Each substrate was supplemented with corn bran and CaCO_3_ at a ratio of 89:10:1 (substrate:corn bran:CaCO_3_) to provide nutrients and regulate pH [9]. Distilled water was added to sawdust-containing substrates to achieve 60–70% moisture content. The substrate compositions are presented in Table 1.

**Figure 1:**
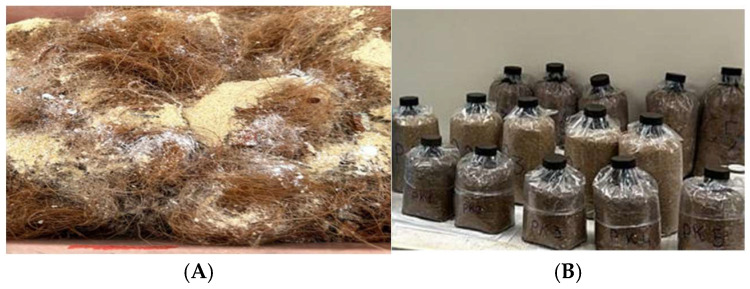
(**A**) Preparation of coconut husk substrate; (**B**) Prepared spawn bags.

The substrates were mixed thoroughly with supplements until homogeneous, then transferred into polyethylene bags (three-quarters full) and capped (Figure 1).

Each substrate treatment was prepared in triplicate. The spawn bags were autoclaved at 121 °C for 15 min and cooled at room temperature for 24 h. Under aseptic conditions in a laminar airflow cabinet, approximately 10 g of *P. pulmonarius* spawn was inoculated into each bag. The sealed bags were incubated in darkness at ambient temperature until complete mycelial colonization occurred.

Following colonization, bags were unsealed and transferred to ambient light conditions to induce fruiting body formation. Substrates were misted twice daily with 1.5–3.0 mL distilled water to maintain moisture levels [9]. Fruiting bodies were harvested manually at maturity, indicated by downward curvature of cap margins. The harvested mushrooms were evaluated for nutritional composition, with sawdust-cultivated mushrooms serving as the control. The fresh samples of mushrooms were dried in a laboratory dryer at 45° Celsius to a constant mass and then ground.

### 2.3. The GC-MS Analysis of Sugars, Phenolic Acid and Fatty Acids

**Figure 2:**
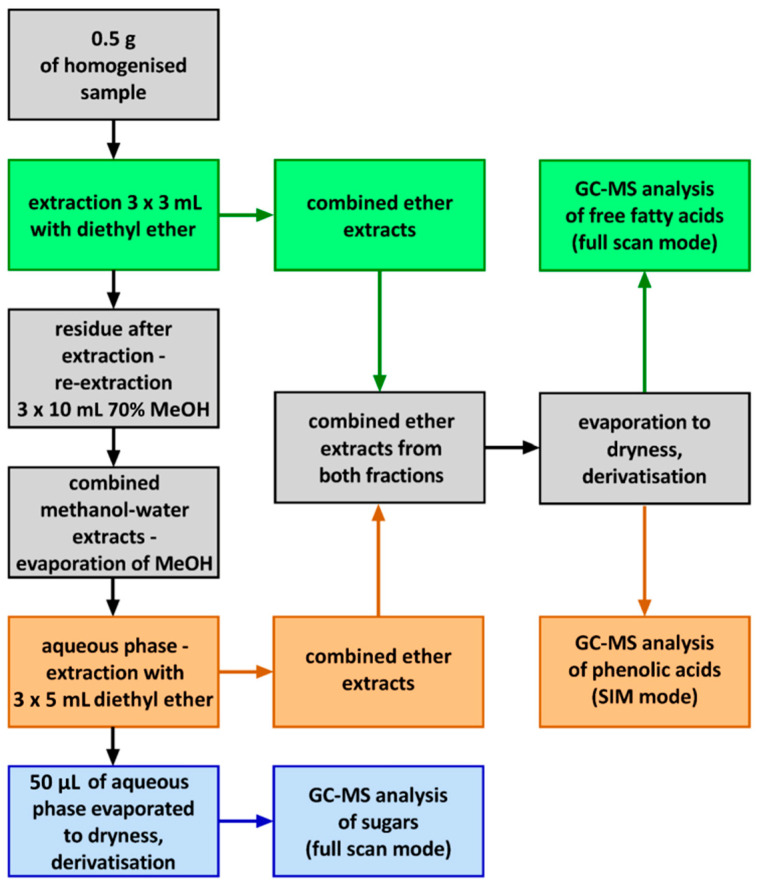
Scheme of GC-MS analysis of sugars, phenolic acid and fatty acids.

A total of 0.5 g of the homogenized sample was extracted three times for 20 min with diethyl ether, each time using 5 mL of solvent. The extracts were combined, dried by adding anhydrous sodium sulfate and evaporated to dryness at 35 °C. The dry residue after extraction was subjected to the derivatization process and analyzed for the content of free fatty acids and phenolic compounds. The material remaining after extraction was extracted three times with 10 mL of 70% methanol. The methanol was evaporated on an evaporator, and 50 µL was taken from the aqueous phase, evaporated to dryness and derivatized for the analysis of sugars. The derivatization process was carried out by adding 250 µL of anhydrous pyridine and 50 µL of BSTFA (bis-trimethylsilane-trifluoroacetamide), then the mixture was heated at 80 °C for 60 min (Figure 2). The solutions prepared in this way were analyzed using a gas chromatograph coupled to a mass spectrometer (GC/MS/MS-Agilent 7890B gas chromatograph coupled with a triple quadrupole mass analyzer Triple Quad 7000C Agilent Technologies), equipped with a split/splitless injector and an HP-5MS capillary column (polydimethylsiloxane with 5% phenyl groups) with dimensions of 30 m × 0.25 mm and a film thickness of 0.25 µm. The analysis parameters were as follows: injector temperature: 260 °C; carrier gas: helium, 6.0 purity; carrier gas flow rate: 1 mL/min (split 10:1); sample injection volume: 1 µL; initial oven temperature: 50 °C; temperature growth rate: 3 °C/min; final oven temperature: 320 °C (10 min. isotherm); transfer line temperature: 300 °C; ion source temperature: 230 °C. The compounds were identified based on the chromatograms of the standard substance using the NIST 2020 Mass Spectral Library and the retention indices of the individual analytes. Under the same conditions of derivatization and chromatographic analysis, measurements of the standard substances of different concentrations were performed and calibration equations were determined, based on which the quantitative analysis was performed.

### 2.4. Metal Content Analysis by ICP-MS

A total of 0.2 g of dried samples (three samples of each type) was weighed and mineralized using 9 mL of 65% nitric acid and 1 mL of 30% peroxide in the Milestone Ethos Easy microwave mineralizer. The temperature program included a temperature increase to 180 °C within 15 min and maintained at 180 °C for 10 min. A blank test was prepared analogously. The cooled mineralizates were filtered, transferred to volumetric flasks, and diluted with deionized water to 25 mL. In the obtained samples, the elements were determined using the 8800 Triple Quadrupole ICP-MS spectrometer (Agilent Technologies). A multi-element standard (Analityk-170 standard solution VIII in 7% HNO_3_, INORGANIC VENTURES) was used to prepare the calibration curves, while 115In (In(NO_3_)_3_ in 2% HNO_3_, Merck) was used as an internal standard. The measurement parameters are presented in Table 2. The content of macroelements (Mg, Ca, Na, K) was determined using the FAAS method. A Thermo Scientific iCE 3000 Series spectrometer with flame atomization was used. The measurement parameters are presented in Table 3.

### 2.5. Total Protein Analysis

A sample of 0.5 g was weighed and placed in a Kjeldahl flask. A total of 20 mL of concentrated 96% sulfuric acid (H_2_SO_4_), 10 mL of 30% hydrogen peroxide (H_2_O_2_), and a catalytic mixture were added. The flask was heated until a clear, colorless solution was obtained. After cooling the solution, alkalization was performed using 30% sodium hydroxide solution (NaOH). The released ammonia was distilled and absorbed in a receiver containing 4% boric acid solution (H_3_BO_3_). The content of absorbed ammonia was titrated using a 0.1 mol/L hydrochloric acid solution (HCl). The analysis was carried out using the FoodALYT D5000 apparatus.

### 2.6. Total Carbohydrates Analysis

The total carbohydrate content was estimated according to the methodology described by Masuko et al. (2005) [10].

### 2.7. FT-IR Analysis

The FTIR spectra of dried fruiting bodies samples were performed using the ATR multi-reflection technique using an Alfa spectrometer (Bruker, Billerica, MA, USA). The spectra were recorded in the range of 2000–600 cm^−1^.

### 2.8. Antioxidant Activity Measurement

#### 2.8.1. Sample Preparation

A total of 0.5 g of the dried sample was mixed with 50 mL of 80% ethanol and subjected to ultrasonic-assisted extraction at 50 °C for 1 h. After extraction, the mixture was filtered to remove solid residues, and the filtrate was collected for further analysis.

#### 2.8.2. DPPH Assay

The free radical scavenging activity was determined using the DPPH assay. A total of 80 μL of the extract samples were transferred into 96-well microplates and adjusted to a final volume of 100 μL with 80% ethanol. Subsequently, 200 μL of a 60 µM DPPH solution prepared in methanol was added to each well. Absorbance was measured at 516 nm using a Tecan Infinite 200 PRO microplate reader (Tecan, Männedorf, Switzerland) [11]. To study the kinetic changes, absorbance readings were taken every 10 min for a total duration of 1 h. A control sample was prepared by replacing the extract with 80% ethanol while maintaining the same reaction conditions. The percentage of DPPH^•^ radical inhibition (%I) was calculated according to the following equation:

(1) %I = (Ac − As)/Ac 100%

where Ac—represents the absorbance of the control sample, As—represents the absorbance of the tested sample.

#### 2.8.3. ABTS Assay

The ABTS^•+^ cation radical solution was generated by mixing a 5.4 mM ABTS solution with 1.74 mM K_2_S_2_O_8_ solution in a 1:1 (*v*/*v*) ratio. The reaction mixture was kept in the darkness for 12 h to ensure formation of the ABTS^•+^. Before use, the resulting solution was diluted with methanol to obtain a working solution with an absorbance of 0.7–0.8 at 734 nm [12]. For antioxidant activity determination, 20 μL of extract was transferred into 96-well microplates and adjusted to a final volume of 100 μL with 80% ethanol. Then 100 µL of the prepared ABTS^•+^ working solution was added to each well. Control samples were prepared simultaneously by substituting the extract with 80% ethanol under identical conditions. The decrease in absorbance was monitored at 734 nm at 60 s intervals over a 14 min period using a Tecan Infinite 200 PRO microplate reader (Tecan, Männedorf, Switzerland). ABTS^•+^ cation radical scavenging activity was expressed as percentage inhibition, calculated according to the same formula applied in the DPPH^•^ assay.

#### 2.8.4. CUPRAC Assay

The cupric ion reducing antioxidant capacity (CUPRAC) of the extracts was evaluated in a 96-well microplate format. Briefly, 20 µL of the tested extract was combined with 180 µL of freshly prepared CUPRAC working reagent. The reagent was composed of 10 mM copper(II) chloride (CuCl_2_), 7.5 mM neocuproine, and ammonium acetate buffer (pH 7.0), mixed in equal proportions (1:1:1, *v*/*v*/*v*) [13]. Absorbance at 450 nm was measured after 60 min using a Tecan Infinite 200 PRO microplate reader (Tecan, Männedorf, Switzerland). Antioxidant capacity was expressed as Trolox equivalents (µM), calculated from a Trolox standard calibration curve (y = 0.7388x + 0.0639; R^2^ = 0.9985).

#### 2.8.5. FRAP Assay

Ferric reducing antioxidant power (FRAP) was assessed by mixing 20 µL of the extract with 180 µL of the FRAP reagent in a 96-well microplate. The FRAP working solution consisted of 10 mM, TPTZ, 20 mM FeCl_3_ ·6H_2_O, and 300 mM acetate buffer pH 3.6 in a ratio 1:1:1 (*v*/*v*/*v*) [14]. The formation of the Fe(II)–TPTZ complex was followed by measuring absorbance at 595 nm after 6 min using a Tecan Infinite 200 PRO microplate reader (Tecan, Männedorf, Switzerland). The ferric-reducing antioxidant activity was quantified as Fe(II) ion equivalents (μM) based on the calibration curve prepared with FeSO_4_ (y = 1.1981x + 0.0574; R^2^ = 0.9998).

All measurements were conducted with five replicates in three independent experiments. The results were expressed as mean ± SD.

#### 2.8.6. Statistical Analysis

One-way analysis of variance (ANOVA) followed by Tukey’s post hoc test was used to assess statistically significant differences among substrate treatment groups. The significance level was set at *p* ≤ 0.05. Principal component analysis (PCA) was performed on a dataset comprising 29 variables representing mineral composition, fatty acid profile, carbohydrate composition, phenolic compounds, total protein content, and antioxidant activity. The analysis was done in program Statistica 13.3PL and Excel 2019 (Microsoft Office).

## 3. Results

### 3.1. The Macro-, Microelements Analysis

The elemental composition of dried fruiting bodies is presented in Table 4. Potassium was the dominant macro-element (19.79–29.04 mg/g), with the highest level in PpS (29.04 mg/g) and the lowest in PpP100 (19.79 mg/g). Magnesium and calcium occurred at comparable levels among mushroom samples (Mg: 0.642–0.714 mg/g; Ca: 0.524–0.691 mg/g), whereas sodium displayed the strongest substrate effect, increasing sharply in PpC (0.615 mg/g) relative to the remaining groups (0.105–0.126 mg/g). Sodium displayed the most pronounced substrate-dependent variation among the macroelements determined, with concentrations in fruiting bodies cultivated on coconut waste (PpC, 0.615 mg/g DW) approximately five-fold higher than those determined for the remaining substrates (PpS: 0.126; PpP20: 0.122; PpP100: 0.105 mg/g DW). This disparity is consistent with the well-documented elevated saline ion content of coconut husk—a material derived from a predominantly coastal plant species which is rich in sodium and chloride [15]. It reflects the established capacity of *Pleurotus* species to bioaccumulate mineral elements directly from their cultivation substrate [16]. Because these macrominerals help adults meet the recommended daily intake of K (3400 mg/day) and Na (<2000 mg/day per WHO guidelines), the notably high K concentrations across PpS, PpP20, and PpC frutting bodies, as well as the elevated Na in PpC, are nutritionally significant [17,18].

Iron and zinc constituted the predominant trace elements in the fruiting bodies of *P. pulmonarius* across all tested substrates, with determined concentrations of 85.50–127.92 µg/g DW and 35.10–54.08 µg/g DW, respectively. Both elements exhibited their highest accumulation in fruiting bodies derived from coconut waste substrate (PpC) and their lowest in those cultivated on paddy husk (PpP100). The observed substrate-dependent variation in trace element content is consistent with the well-documented capacity of *Pleurotus* species to bioaccumulate minerals from their growth medium, and may be attributed to differences in the inherent mineral profiles of the respective substrates. All values for potentially toxic elements were well within the acceptable ranges set by the China National Food Safety Standard GB 2762-2022 (Cd: 0.5 mg/kg; Pb: 1.0 mg/kg; Cr: 1.0 mg/kg) [19], with the exception of chromium which recorded 4times the permissible levels in the fruiting bodies from all four substrates. The highest levels of cadmium were found in PpS (0.725 µg/g), while the lowest levels were found in PpC and PpP20 (~0.134–0.138 µg/g), and the highest levels of lead were found in PpP100 (0.753 µg/g). The well-established function of metallothionein, a metal-binding protein frequently present in edible mushrooms, which selectively binds and sequesters heavy metals, facilitating their bioaccumulation from the substrate [20], is consistent with the accumulation of these potentially hazardous elements in *P. pulmonarius* fruiting bodies. While traditional cooking techniques like stewing, grilling, and frying have been demonstrated to partially reduce mineral content [21], the potential buildup of heavy metals in fruiting bodies warrants ongoing monitoring, especially when cultivation substrates of variable or uncertain mineral composition are used. Commercial mushroom growers should analyze the substrates used in the cultivation of mushrooms for heavy metal content prior to usage, to minimize the risk of heavy metal bioaccumulation in the fruiting bodies. Furthermore, total Cr is often used to assess Cr risk from food consumption which may overestimate its health risk because not all Cr in mushrooms is bioavailable [22].

The obtained data demonstrate that the chosen substrates can substantially re-shape the mineral fingerprint of oyster mushrooms, particularly for sodium and key trace elements (iron, zinc, nickel, chromium), while maintaining the characteristic potassium-rich profile.

### 3.2. GC-MS Analysis of Fatty Acids, Carbohydrates and Phenolic Compound

#### 3.2.1. Fatty Acid Profile

GC-MS analysis revealed that unsaturated fatty acids predominated over saturated fatty acids in all mushroom samples, which is characteristic of *Pleurotus* species and nutritionally desirable. Linoleic acid was the dominant fatty acid in all mushroom samples. Its concentration was markedly higher in PpS (2.411 mg/g DW) compared to PpC (0.598 mg/g DW), PpP20 (0.675 mg/g DW), and PpP100 (0.690 mg/g DW) (Table 5). A similar trend was observed for oleic acid and stearic acid, both of which were highest in the control (PpS). Palmitic acid content was also substantially higher in PpS (0.436 mg/g DW) than in the other mushroom samples (0.117–0.133 mg/g DW). In *P. ostreatus* the lipid fraction is typically dominated by linoleic acid (C18:2), followed by oleic (C18:1), palmitic (C16:0) and stearic (C18:0) acids which are widely described as characteristic for edible mushrooms and oyster mushrooms in particular [23]. Our results show exactly this qualitative pattern, with linoleic acid as the major fatty acid in all samples, and oleic/palmitic/stearic acids as the next most abundant [24,25]. From a nutritional perspective, the high proportion of linoleic acid (an essential omega-6 fatty acid) enhances the dietary value of the mushrooms, as this fatty acid plays an important role in membrane structure and human metabolic processes.

The sample grown on sawdust (PpS) contained markedly higher levels of the main fatty acids (especially linoleic and oleic acids) than mushrooms cultivated on coconut or paddy-based wastes. It suggests that the sawdust substrate (PpS) promotes lipid biosynthesis more effectively than other waste substrates used in this study.

#### 3.2.2. Total Carbohydrate Analysis

The total carbohydrate content of dried mushroom samples is presented in Table 6. Carbohydrate levels ranged from 297.35 to 459.97 mg/g. Reported total carbohydrate contents for oyster mushrooms typically range between 35 and 60% DW, depending on substrate, strain, and analytical method [5]. Comprehensive nutritional reviews indicate that carbohydrates represent the major macronutrient fraction in oyster mushrooms, primarily in the form of structural polysaccharides (β-glucans, chitin), storage sugars (trehalose), and sugar alcohols [26]. The values obtained in the present study (29.7–46.0% DW) fall within the lower-to-middle portion of the range commonly reported for other *Pleurotus* species.

The highest carbohydrate concentration was observed in PpP100 (459.97 mg/g DW), followed by PpP20 (420.06 mg/g DW) and PpC (404.92 mg/g DW), whereas the lowest value was recorded in the control sample PpS (297.35 mg/g DW). These results clearly demonstrate that agragrii-substrates, particularly paddy husk media, promote greater carbohydrate accumulation in mushrooms compared to conventional sawdust. The elevated carbohydrate levels in PpP100 and PpP20 are consistent with the GC–MS findings showing increased trehalose content in these samples, suggesting enhanced carbon storage metabolism under these cultivation conditions. The relatively lower carbohydrate content in the sawdust-grown mushroom (PpS) may reflect a metabolic shift toward lipid accumulation, as evidenced by the higher fatty acid content observed in that sample. The literature further confirms that substrate composition strongly influences carbohydrate accumulation in *Pleurotus* spp. samples due to differences in lignocellulosic composition, carbon availability, and C:N ratio [16,27]. Therefore, the increased carbohydrate content observed in mushrooms cultivated on paddy husk substrates is consistent with previously documented substrate-driven modulation of fatty acid synthesis. Overall, the results indicate that agri-waste substrates not only maintain but can enhance carbohydrate accumulation in oyster mushrooms, supporting their suitability for sustainable production of carbohydrate-rich functional foods. The incorporation of mushrooms into breads has been discussed by Lu et al. (2025) as a practical way to increase the nutritional benefits of these bioactive-rich breads by increasing the presence of slowly digestible starches (SDSs) which are know to have lower glycemic indices [28].

GC-MS analysis was performed to elucidate more specifically the carbohydrate content in these samples (Table 7). Among the carbohydrate moieties detected, trehalose was the predominant carbohydrate in all samples, confirming its role as the principal storage sugar in oyster mushrooms. The highest trehalose content was observed in: PpP20 (101.015 mg/g DW), followed by PpC (96.985 mg/g DW), PpP100 (84.223 mg/g DW), and PpS (72.769 mg/g DW) (Table 7). Thus, mushrooms grown on paddy husk supplemented with sawdust (PpP20) accumulated the highest amount of this protective disaccharide [28]. Fructose was the second most abundant simple sugar, with the highest concentration detected in PpS (2.012 mg/g), whereas the lowest was found in PpP100 (0.865 mg/g). Interestingly, myo-inositol content was markedly elevated in PpC (3.498 mg/g), significantly exceeding the levels in other treatments. This finding may be potentially linked to the simultaneously elevated concentrations of metals (including Fe, Ni, and Zn) recorded in PpC fruiting bodies, given the well-documented relationship between inositol signaling and metal stress responses in fungi [29].

The literature data showed that in *P. ostreatus* the soluble carbohydrates are largely composed of trehalose, mannitol, and glucose (often with smaller contributions of other sugars/sugar alcohols), and that their levels change with developmental stage and cultivation conditions [30,31].

#### 3.2.3. Phenolic Compound and Sterols Profile

Phenolic acids were detected in all samples, though their concentrations varied considerably depending on substrate type. The most striking result was the extremely high cinnamic acid content in PpS (10.550 µg/g DW), whereas in other samples it ranged only between 0.779 and 1.067 µg/g DW (Table 8). Similarly, *p*-coumaric acid and 4-hydroxybenzeneacetic acid were significantly higher in PpS compared to other treatments. However, some phenolics were elevated in agro-waste substrates. For example: vanillic acid was notably higher in PpC (0.376 µg/g), protocatechuic acid was the highest in PpC (0.208 µg/g), and other minor phenolics were also substrate-dependent (Supplementary File: Tables S1 and S2). PpS exhibited the highest cumulative concentration of several key phenolic acids, suggesting stronger antioxidant potential linked to aromatic acid biosynthesis. Nevertheless, coconut waste (PpC) enhances specific antioxidant-related compounds such as vanillic and protocatechuic acids.

The literature data showed that in *P. ostreatus* from both benzoic-acid derivatives (e.g., protocatechuic, vanillic, 4-hydroxybenzoic) and cinnamic-acid derivatives were identified (e.g., *p*-coumaric, ferulic, trans-cinnamic) [32,33]. The phenolic spectrum detected in our GC-MS dataset for *P. pulmonarius* (Supplementary File: Tables S1 and S2)—particularly cinnamic acid, *p*-coumaric acid, protocatechuic acid, and vanillic acid—is therefore fully consistent with the phenolic “fingerprint” described for *P. ostreatus*. However, certain phenolic acids, such as vanillic and protocatechuic acids, were relatively elevated in the coconut waste substrate (PpC), indicating that the type of agro-waste may selectively stimulate specific branches of phenolic metabolism.

Comparative literature data for *P. ostreatus* confirm that phenolic acid content is highly variable and influenced by substrate composition, extraction method and analytical technique. The phenolic concentrations reported in different studies often vary due to methodological differences (e.g., HPLC vs. GC-MS after derivatization), which should be considered when making direct numerical comparisons. Nevertheless, the qualitative agreement in phenolic classes and the dominance of cinnamic/benzoic derivatives confirm that the studied mushrooms exhibit a metabolic pattern characteristic of *Pleurotus* species.

The total GC-MS data clearly demonstrate that substrate composition significantly influences secondary metabolism in *P. pulmonarius*. Sawdust (PpS) favored lipid and phenolic acid accumulation. Paddy husk supplementation (PpP20) enhanced trehalose accumulation. Coconut waste (PpC) promoted inositol and selected phenolic biosynthesis. Pure paddy husk (PpP100) showed intermediate metabolic profiles. These findings confirm that agri-wastes can be successfully used as alternative substrates without compromising nutritional quality, while in some cases even enhancing specific nutraceutical compounds.

GC-MS analysis of the ethyl acetate extract also revealed the presence of fungal sterols, with ergosterol being the dominant sterol fraction; its relative content varied significantly depending on the substrate, reaching the highest levels in PpP20 (15.89%) and PpC (13.07%), while much lower amounts were detected in PpP100 (2.92%) and PpS (0.65%). Dehydroergosterol and ergosterol peroxide were additionally identified in all samples, with the latter showing the highest relative content in PpS (2.32%), suggesting substrate-dependent modulation of sterol biosynthesis and oxidative conversion pathways in *P. pulmonarius*.

### 3.3. Total Protein Analysis

Protein levels ranged from 173.77 to 223.30 mg/g DW, corresponding to 17.4–22.3% of dry weight (Table 9). The highest protein content was recorded in mushrooms cultivated on coconut waste (PpC, 223.30 mg/g DW), followed by paddy husk 100% (PpP100, 204.42 mg/g DW). Lower but comparable values were observed for PpP20 (174.36 mg/g DW) and the control PpS (173.77 mg/g DW). These findings clearly indicate a substrate-dependent modulation of protein accumulation, with agri-waste (especially coconut waste) increasing protein biosynthesis compared to conventional sawdust. Such relationships are widely attributed to differences in substrate C:N ratio, nitrogen bioavailability, and mineral composition, which directly influence the efficiency of fungal nitrogen assimilation and enzymatic protein synthesis. Protein contents for *Pleurotus ostreatus* typically range between 15 and 30% DW, depending on strain, cultivation conditions, and nitrogen conversion factor applied [5,34].

The protein levels obtained in the present study for *P. Pulmonarius* (17.4–22.3% DW) fall within the upper-middle range of these literature values, confirming that cultivation on agro-wastes maintains the protein nutritional quality relative to commonly reported data for *P. ostreatus*. Similar substrate-induced modulation of protein accumulation has been widely documented in oyster mushrooms cultivated on alternative lignocellulosic agro-wastes, with protein content consistently varying as a function of substrate nitrogen availability and C/N ratio [16,27]. The obtained results confirm that coconut and paddy husk wastes are effective sustainable substrates capable of producing protein-rich *P. pulmonarius* with nutritional values fully comparable to those documented for *Pleurotus* spp.

### 3.4. FTIR Analysis

**Figure 3:**
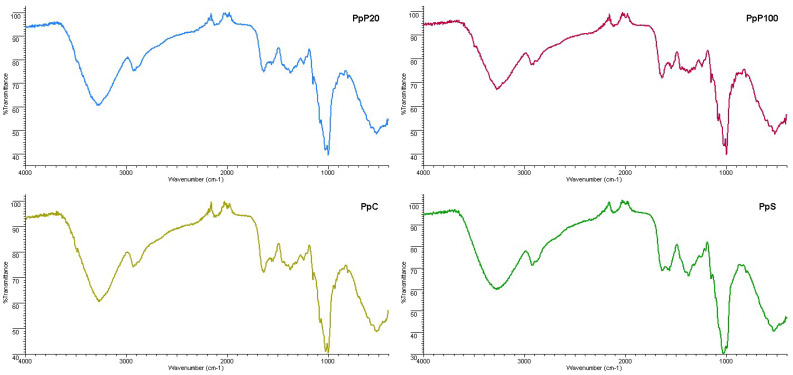
FTIR spectra of dried oyster mushroom samples.

Figure 3 shows the FTIR spectra of dried oyster mushrooms (PpP20, PpP100, PpC and PpS) recorded in the range of 3600–400 cm^−1^. The assignment of the bands present in the spectra is summarized in Table 10.

Characteristic bands in the spectra of the studied oyster mushroom species were observed, indicating the presence of polysaccharides, fats, and proteins. An intense, broad band located at approximately 3270 cm^−1^ originates from the stretching vibrations of the OH and NH groups, indicating the presence of polysaccharides, including chitin, and proteins [4]. In the wavenumber range of 2932–2872 cm^−1^, bands associated with the stretching vibrations of the CH_2_ and CH_3_ groups are located, indicating the presence of fats (including fatty acids), saccharides, and proteins [35]. A medium-intensity band located at approximately 1635 cm^−1^ is the amide I band, associated with the stretching vibrations of the carbonyl group [36]. The presence of this band indicates the content of amino acids and carboxylic acids, including aromatic acids. The presence of carboxylic acids in the tested samples is also indicated by bands located in the spectra at wavenumbers 1373–1309 cm^−1^. These bands originate from vibrations of the carboxylate anion. In the wavenumber range 1251 cm^−1^ to 1147 cm^−1^, the amide II bands and the stretching vibration bands of the P-O group are located [37,38]. This confirms the presence of polysaccharides, proteins, and phosphorus esters in the dried mushrooms [4]. Bands originating from C-H ring deformation vibrations, present in the wavenumber range 932–831 cm^−1^, are associated with the presence of glucans and chitin in the samples and may also originate from aromatic compounds [39,40]. In the wavenumber range 606–518 cm^−1^, bands associated with vibrations of groups present in mineral compounds are located.

Comparing the spectra of dried mushrooms grown on different media, it can be concluded that each sample contains polysaccharide, protein, and lipid fractions, as well as aromatic and mineral compounds. No significant differences were observed between the spectra of the tested samples. The absence of bands at approximately 1078 cm^−1^ and 930 cm^−1^ in the spectrum of the dried PpS sample may indicate a slightly lower glucan content in this sample compared to the other dried mushrooms.

### 3.5. Antioxidant Activity Measured in DPPH, ABTS, CUPRAC and FRAP Assay

#### 3.5.1. DPPH Assay

**Figure 4:**
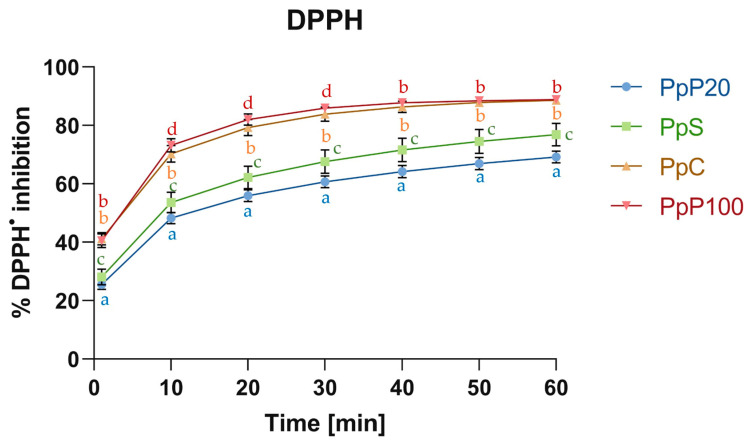
Kinetics of the DPPH radical scavenging reaction. The results represent the mean values from three independent experiments, each performed in six replicates. Standard deviations (SDs) are indicated on the graphs. Statistical differences among substrate groups were evaluated at each time point using one-way ANOVA followed by Tukey’s post hoc test (*p* ≤ 0.05). Different colored letters above data points indicate statistically significant differences at the corresponding time point (*p* ≤ 0.05). Colored letters correspond to the corresponding kinetic curves.

The antioxidant activity of *Pleurotus pulmonarius* fruiting bodies cultivated on four different substrates was evaluated using the DPPH^•^ radical scavenging assay. Ethanolic extracts (80% EtOH) were tested with kinetic measurements recorded every 10 min over 60 min (Figure 4). The kinetic profiles show the most rapid radical scavenging within the first 10–20 min, followed by a gradual plateau reached between 40 and 60 min. This is a pattern characteristic of mixed HAT/SPLET reaction antioxidant mechanisms [41,42]. After 60 min, PpP100 and PpC showed the greatest inhibition of DPPH^•^, reaching 88.85 ± 0.26% and 88.59 ± 0.74%, respectively, with no statistically significant difference between them. These values clearly exceeded those of PpS (76.91 ± 3.89%) and PpP20 (69.17 ± 2.07%), resulting in a statistically significant difference in activity, which can be ranked PpP100 ≈ PpC > PpS > PpP20.

These results indicate that substrate composition significantly influences the accumulation of antioxidant compounds in *P. pulmonarius* fruiting bodies. The superior activity observed in PpP100 and PpC may be attributed to the lignocellulosic composition of rice and coconut residues, which may act as precursors or inducers of phenolic biosynthetic pathways, or provide enhanced mineral availability (e.g., Mn as a cofactor for laccase and manganese peroxidases). Conversely, the dominance of sawdust in the PpP20 substrate appears to suppress the accumulation of relevant secondary metabolites, resulting in the lowest antioxidant activity. Overall, the data demonstrate that cultivation on agricultural by-products, particularly paddy husk and coconut waste, can substantially enhance the antioxidant potential of *P. pulmonarius*, supporting their use as value-added substrates in functional mushroom production.

#### 3.5.2. ABTS Assay

**Figure 5:**
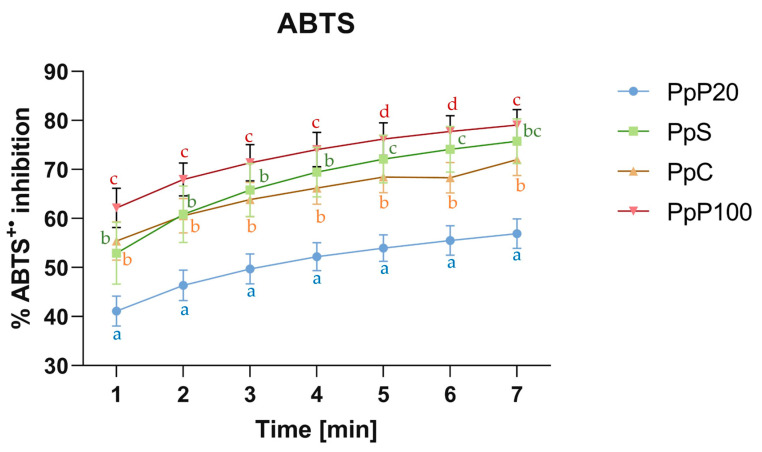
Kinetics of the ABTS radical scavenging reaction. The results represent the mean values from three independent experiments, each performed in six replicates. Standard deviations (SDs) are indicated on the graphs. Statistical differences among substrate groups were evaluated at each time point using one-way ANOVA followed by Tukey’s post hoc test (*p* ≤ 0.05). Different colored letters above data points indicate statistically significant differences at the corresponding time point (*p* ≤ 0.05). Colored letters correspond to the corresponding kinetic curves.

The ABTS^•+^ radical scavenging activity of *P. pulmonarius* extracts was assessed over a 7 min kinetic measurement period recorded at 1 min intervals (Figure 5). Notably, compared to the DPPH assay, the ABTS reaction reached near-plateau conditions much faster (within the first 3–4 min) which is consistent with the higher reactivity of the ABTS^•+^ cation radical and the predominantly electron-transfer (SET) mechanism governing this assay [42,43]. Inhibition of ABTS^•+^ increased substantially across all variants, with PpP100 reaching the highest value of 79.10 ± 3.17%, followed by PpC (72.00 ± 3.28%), PpS (75.79 ± 4.55%), and Pp20 (56.91 ± 3.04%). The statistically significant activity ranking was therefore PpP100 > PpS > PpC > PpP20.

The higher antioxidant activity of PpP100 extract across both assays supports the conclusion that cultivation on paddy husk substrate promotes the accumulation of antioxidant compounds, active via both hydrogen atom transfer and electron transfer mechanisms.

#### 3.5.3. CUPRAC and FRAP Assays

**Figure 6:**
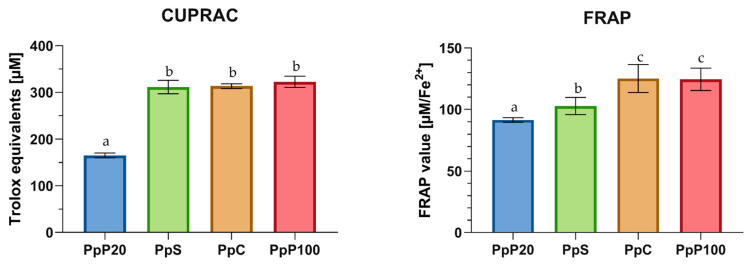
Antioxidant activity of *Pleurotus pulmonarius* fruiting body extracts cultivated on different substrates determined by the CUPRAC assay (expressed as Trolox equivalents) and FRAP assay (expressed as Fe^2+^ equivalents). Values represent ±SD of three independent experiments; each performed on six replicates. Different lowercase letters above the bars indicated statistically significant differences among substrates groups (*p* ≤ 0.05, one ANOVA, Tukey’s post hoc test).

The CUPRAC assay yielded Trolox equivalent values of 322.58 ± 12.16 µM (PpP100), 313.36 ± 5.19 µM (PpC), 311.56 ± 14.40 µM (PpS), and 165.28 ± 5.13 µM (PpP20), establishing an activity ranking of PpP100 ≈ PpC ≈ PpS > PpP20 (Figure 6). The three strongest samples were nearly indistinguishable from one another, with differences falling within the range of their respective standard deviations, whereas PpP20 exhibited reducing capacity approximately half that of the remaining variants.

The FRAP assay quantifies antioxidant capacity based on the ability of sample components to reduce ferric ions (Fe^3+^) to ferrous ions (Fe^2+^) in the presence of TPTZ ligand, with results expressed as Fe^2+^ equivalents (µM Fe^2+^). Values recorded after 6 min of reaction were 116.36 ± 10.83 µM Fe^2+^ (PpC), 115.40 ± 8.51 µM Fe^2+^ (PpP100), 98.01 ± 6.64 µM Fe^2+^ (PpS), and 85.47 ± 1.67 µM Fe^2+^ (PpP20), yielding a statistically significant activity ranking of PpC ≈ PpP100 > PpS > PpP20.

The FRAP and CUPRAC assays both measure antioxidant capacity through electron transfer-based reduction of a metal ion complex (Fe^3+^/TPTZ in FRAP and Cu^2+^/neocuproine in CUPRAC), but they operate under markedly different pH conditions, FRAP at pH 3.6 (acetate buffer) and CUPRAC at pH 7.0 (ammonium acetate buffer), which directly influences the ionization state and redox potential of antioxidant compounds present in the extracts. Compounds with higher pKa values, such as phenolic acids and organic acids identified by GC-MS in the tested extracts, are predominantly protonated and thus more reactive as electron donors under the acidic conditions of the FRAP assay, whereas the near-physiological pH of the CUPRAC system favors deprotonated. This difference may account for the observed shifts in relative sample ranking between the two methods. Differences in the results obtained for the same samples depending on the method used have been noted and described in our other works. This is a result of different reaction mechanisms and the pH of the reaction medium during the assay [44,45].

## 4. PCA

**Figure 7:**
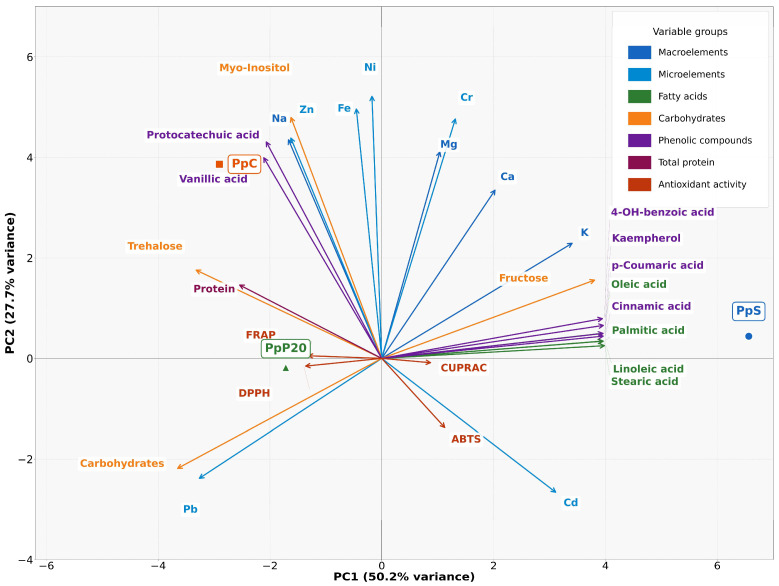
PCA biplot of *Pleurotus pulmonarius* fruiting bodies cultivated on four agro-waste substrates. PC1 and PC2 explain 50.2% and 27.7% of total variance, respectively. Arrows represent variable loadings; colors indicate variable groups as shown in the legend.

PCA was applied to the dataset of 29 variables covering mineral composition, fatty acids, carbohydrates, phenolic acids, total protein, and antioxidant activity. PC1 and PC2 together accounted for 78.0% of the total variance (50.2% and 27.7%, respectively), and the resulting biplot showed clear separation among all four substrate groups (Figure 7). The position of PpS far along the positive PC1 axis reflected its markedly higher fatty acid content (linoleic, oleic, palmitic, and stearic acids all loaded strongly in this direction) and phenolic acids, particularly cinnamic and *p*-coumaric acids. This component effectively separated PpS from the remaining substrates, confirming that sawdust cultivation strongly promotes lipid and phenolic acid biosynthesis in *P. pulmonarius*. The negative region of PC1 was associated with carbohydrate-related variables (total carbohydrates, trehalose), consistent with the higher carbohydrate content observed in paddy husk-based substrates (PpP100, PpP20). PC2 was dominated by microelements (Fe, Zn, Ni, Cr) and macroelements (Na, Mg), which displayed high positive loadings, separating PpC distinctly from PpP100 along this axis. This pattern reflects the mineral-rich character of coconut waste substrate and its capacity to promote bioaccumulation of trace elements in mushroom fruiting bodies. Specific phenolic acids associated with coconut waste cultivation, namely vanillic and protocatechuic acids, also contributed positively to PC2, further reinforcing the unique nutraceutical fingerprint of PpC. Conversely, PpP100 was positioned in the negative region of PC2, consistent with its highest carbohydrate accumulation and lowest mineral content among the tested substrates.

## 5. Conclusions

This study demonstrates that cultivation substrate significantly influences the nutritional composition, secondary metabolite profile, and antioxidant properties of *Pleurotus pulmonarius* fruiting bodies. Among the tested substrates, coconut waste (PpC) promoted the highest protein accumulation (223.30 mg/g DW) and enhanced iron, zinc, and specific phenolic acid biosynthesis (vanillic, protocatechuic acids), while paddy husk-based substrates (PpP100, PpP20) favored carbohydrate accumulation, particularly trehalose. The sawdust control (PpS) supported the greatest lipid content, especially linoleic acid, reflecting a substrate-driven metabolic reallocation between lipid and protein biosynthesis. These findings were corroborated by FTIR analysis, which confirmed the presence of polysaccharide, protein, and lipid fractions in all samples, with a slight reduction in glucan-associated bands observed in PpS. Potassium is the dominant macroelement across all substrates, with PpS recording the highest K content (29.04 mg/g DW). Iron and zinc were the most abundant trace elements, both peaking in PpC (127.92 and 54.08 µg/g DW, respectively), indicating enhanced trace-metal bioavailability under coconut waste cultivation. Antioxidant assessment using four complementary assays consistently identified PpP100 and PpC as the most active mushrooms, with PpP20 recording the lowest activity regardless of the mechanistic basis of the assay. Overall, the results confirm that agro-waste substrates maintain or enhance the nutritional and nutraceutical quality of oyster mushrooms, supporting their use as sustainable and value-added alternatives to conventional sawdust in *Pleurotus* cultivation. The PCA demonstrates that each cultivation substrate generates a biochemically distinct profile in *P. pulmonarius* fruiting bodies. The clear separation of all four groups confirms the strong substrate-driven modulation of nutritional composition, secondary metabolite profiles, and antioxidant properties reported in the individual analytical results.

## Figures and Tables

**Table 1:** Substrate composition used for the cultivation of *Pleurotus pulmonarius*.

Substrate	Name of the *P. pulmonarius* Samples Growing on a Given Substrate	Substrate [kg]	Corn Bran [g]	CaCO_3_ [g]	Added Water [L]
Paddy husk 100%	PpP100	2.2	220	22	Pre-soaked
Paddy husk 20% + Sawdust 80%	PpP20	2.2	220	22	3.0
Coconut waste 100%	PpC	2.0	200	22	Pre-soaked
Sawdust 100% (control)	PpS	2.0	200	22	3.0

**Table 2:** ICP-MS measurement conditions.

Parameter	Conditions		
RF power	1550 W		
Plasma gas flow	15 L/min		
Auxillary gas flow	0.9 L/min		
Nebulizer gas flow	1.05 L/min		
Sample introduction system	Glass concentric MicroMist nebulizer, quartz Scott-type spray chamber, quartz torch with 2.5 mm injector		
Sample inlet flow	0.35 mL/min		
Plasma mode	General purpose		
Cell gas mode (flow rate)	He (5 mL/min)	High Energy He (10 mL/min)	O_2_ (0.4 mL/min)
Scan type	Single Quad	MS/MS	MS/MS
Monitored isotopes	9 Be, 23 Na, 24 Mg, 27 Al, 39 K, 44 Ca, 55 Mn, 59 Co, 60 Ni, 63 Cu, 66 Zn, 88 Sr, 111 Cd, 135 Ba, 205 Tl, 208 Pb, 209 Bi	56 → 56 Fe	52 → 68 Cr 28 → 44 Si

**Table 3:** FAAS measurement conditions.

	Wavelength (nm)	Width of the Gap (nm)	Gas Flow (Acetylene, Air) (L/min)	Lamp Current (mA)
Ca	422.7	0.5	1.4	6
Mg	285.2	0.5	1.0	4
K	766.5	0.5	1.2	8
Na	589.0	0.2	1.1	8

**Table 4:** The content of metals in dried weight (DW) of mushroom samples.

		PpS	PpC	PpP20	PpP100
Mg	mg/g	0.695 ± 0.002 ^a^	0.714 ± 0.009 ^b^	0.642 ± 0.004 ^c^	0.653 ± 0.007 ^d^
Ca	mg/g	0.691 ± 0.003 ^a^	0.681 ± 0.001 ^b^	0.524 ± 0.002 ^c^	0.565 ± 0.001 ^d^
Na	mg/g	0.126 ± 0.001 ^a^	0.615 ± 0.002 ^b^	0.122 ± 0.002 ^c^	0.105 ± 0.001 ^d^
K	mg/g	29.043 ± 0.203 ^a^	23.103 ± 0.023 ^b^	24.398 ± 0.293 ^c^	19.790 ± 0.100 ^d^
Cr	µg/g	4.600 ± 0.037 ^a^	4.683 ± 0.066 ^b^	4.044 ± 0.069 ^c^	0.433 ± 0.003 ^d^
Fe	µg/g	109.206 ± 0.983 ^a^	127.917 ± 1.535 ^b^	120.620 ± 0.724 ^c^	85.503 ± 0.513 ^d^
Ni	µg/g	1.835 ± 0.040 ^a^	2.879 ± 0.095 ^b^	2.133 ± 0.036 ^c^	0.330 ± 0.011 ^d^
Zn	µg/g	40.426 ± 0.283 ^a^	54.075 ± 0.270 ^b^	51.801 ± 0.259 ^c^	35.096 ± 0.632 ^d^
Cd	µg/g	0.725 ± 0.017 ^a^	0.134 ± 0.004 ^b^	0.138 ± 0.004 ^b^	0.548 ± 0.016 ^c^
Pb	µg/g	0.527 ± 0.005 ^a^	0.667 ± 0.007 ^b^	0.625 ± 0.004 ^c^	0.753 ± 0.002 ^d^

Values marked with different superscript letters (a, b, c, d) within the same row differ significantly (*p* ≤ 0.05). Data are expressed as mean ± standard deviation (SD). Statistical significance among samples was assessed by one-way analysis of variance (ANOVA) followed by Tukey’s honestly significant difference (HSD) post hoc test, using Statistica 13.3 PL software.

**Table 5:** The content of fatty acids in dried weight of mushroom samples determined by GC-MS method.

mg/g	PpS	PpC	PpP20	PpP100
Tetradecanoic acid	0.019 ± 0.0005 ^a^	0.006 ± 0.0002 ^b^	0.008 ± 0.0001 ^c^	0.007 ± 0.0002 ^d^
Pentadecanoid acid	0.051 ± 0.0009 ^a^	0.027 ± 0.0008 ^b^	0.013 ± 0.0004 ^c^	0.033 ± 0.0004 ^d^
Palmitoleic acid	0.016 ± 0.0004 ^a^	0.011 ± 0.0003 ^b^	0.010 ± 0.0001 ^c^	0.013 ± 0.0004 ^d^
Palmitic Acid	0.436 ± 0.0078 ^a^	0.123 ± 0.0024 ^b^	0.117 ± 0.0034 ^c^	0.133 ± 0.0015 ^d^
Linoleic acid	2.411 ± 0.0239 ^a^	0.598 ± 0.0062 ^b^	0.675 ± 0.0052 ^c^	0.690 ± 0.0070 ^d^
Oleic acid	0.777 ± 0.0145 ^a^	0.199 ± 0.0021 ^b^	0.174 ± 0.0048 ^c^	0.197 ± 0.0052 ^b^
Stearic acid	0.380 ± 0.0076 ^a^	0.070 ± 0.0025 ^b^	0.054 ± 0.0016 ^c^	0.080 ± 0.0028 ^d^

Values marked with different superscript letters (a, b, c, d) within the same row differ significantly (*p* ≤ 0.05). Data are expressed as mean ± standard deviation (SD). Statistical significance among samples was assessed by one-way analysis of variance (ANOVA) followed by Tukey’s honestly significant difference (HSD) post hoc test, using Statistica 13.3 PL software.

**Table 6:** Content of total carbohydrates in dried weight of mushroom samples.

Sample	Total Carbohydrate Content [mg/g]
PpP100	459.97 ± 21.69 ^a^
PpS	297.35 ± 4.27 ^b^
PpC	404.92 ± 11.98 ^c^
PpP20	420.06 ± 25.21 ^d^

Values marked with different superscript letters (a, b, c, d) within the same row differ significantly (*p* ≤ 0.05). Data are expressed as mean ± standard deviation (SD). Statistical significance among samples was assessed by one-way analysis of variance (ANOVA) followed by Tukey’s honestly significant difference (HSD) post hoc test, using Statistica 13.3 PL software.

**Table 7:** The content of carbohydrates in dried weight of mushroom samples determined by GC-MS method.

mg/g	PpS	PpC	PpP20	PpP100
Fructose	2.012 ± 0.0971 ^a^	1.116 ± 0.0597 ^b^	1.158 ± 0.0290 ^b^	0.865 ± 0.0197 ^c^
Glucose	0.318 ± 0.0088 ^a^	0.266 ± 0.0094 ^b^	0.159 ± 0.0034 ^c^	0.078 ± 0.0011 ^d^
Sucrose	0.575 ± 0.0196 ^a^	0.750 ± 0.0212 ^b^	0.585 ± 0.0188 ^a^	0.532 ± 0.0140 ^c^
Trehalose	72.769 ± 0.7937 ^a^	96.985 ± 1.0166 ^b^	101.015 ± 1.7838 ^c^	84.223 ± 0.9876 ^d^
Chiro-Inositol	0.385 ± 0.0023 ^a^	0.041 ± 0.0004 ^b^	0.025 ± 0.0005 ^c^	0.032 ± 0.0002 ^d^
Scyllo-Inositol	0.432 ± 0.0022 ^a^	0.628 ± 0.0063 ^b^	0.036 ± 0.0003 ^c^	0.033 ± 0.0006 ^c^
Myo-Inositol	0.657 ± 0.0084 ^a^	3.498 ± 0.0705 ^b^	1.064 ± 0.0078 ^c^	0.151 ± 0.0016 ^d^

Values marked with different superscript letters (a, b, c, d) within the same row differ significantly (*p* ≤ 0.05). Data are expressed as mean ± standard deviation (SD). Statistical significance among samples was assessed by one-way analysis of variance (ANOVA) followed by Tukey’s honestly significant difference (HSD) post hoc test, using Statistica 13.3 PL software.

**Table 8:** The content of phenolic compounds in dried weight of mushroom samples determined by GC-MS method. Values marked with different superscript letters (a, b, c, d) within the same row differ significantly (*p* ≤ 0.05). Data are expressed as mean ± standard deviation (SD). Statistical significance among samples was assessed by one-way analysis of variance (ANOVA) followed by Tukey’s honestly significant difference (HSD) post hoc test, using Statistica 13.3 PL software.

µg/g	PpS	PpC	PpP20	PpP100
Cinnamic acid	10.550 ± 0.2807 ^a^	0.978 ± 0.0128 ^b^	0.779 ± 0.0123 ^c^	1.067 ± 0.0154 ^b^
3-Hydroxybenzeneacetic acid	0.007 ± 0.0000 ^a^	0.013 ± 0.0001 ^b^	0.009 ± 0.0001 ^c^	0.013 ± 0.0001 ^b^
Vanillic acid	0.018 ± 0.0005 ^a^	0.376 ± 0.0023 ^b^	0.053 ± 0.0004 ^c^	0.055 ± 0.0004 ^d^
4-Hydroxybenzeneacetic acid	2.318 ± 0.0443 ^a^	0.237 ± 0.0020 ^b^	0.225 ± 0.0014 ^b^	0.085 ± 0.0013 ^c^
Protocatechuic acid	0.055 ± 0.0002 ^a^	0.208 ± 0.009 ^b^	0.080 ± 0.0010 ^c^	0.059 ± 0.0008 ^d^
*p *-Coumaric acid	2.423 ± 0.0368 ^a^	0.159 ± 0.009 ^b^	0.109 ± 0.0007 ^c^	0.152 ± 0.0012 ^b^
Gallic acid	0.017 ± 0.0008 ^a^	0.006 ± 0.0001 ^b^	0.006 ± 0.0001 ^b^	0.004 ± 0.0002 ^c^
Kaempherol	0.474 ± 0.0078 ^a^	0.054 ± 0.0006 ^b^	0.072 ± 0.0009 ^c^	0.037 ± 0.0004 ^d^

**Table 9:** The total content of protein in dried weight of mushroom samples.

Sample	Protein Content [mg/g]
PpS	173.77 ± 0.452 ^a^
PpC	223.30 ± 1.541 ^b^
PpP 100	204.42 ± 0.388 ^c^
PpP 20	174.36 ± 0.593 ^d^

Values marked with different superscript letters (a, b, c, d) within the same row differ significantly (*p* ≤ 0.05). Data are expressed as mean ± standard deviation (SD). Statistical significance among samples was assessed by one-way analysis of variance (ANOVA) followed by Tukey’s honestly significant difference (HSD) post hoc test, using Statistica 13.3 PL software.

**Table 10:** FTIR band assignments of dried *P. pulmonarius* samples.

PpP20	PpP100	PpC	PpS	Type of Vibration	Groups of Chemical Compounds
Wavenumber [cm^−1^] and Intensities					
3267 s	3271 m	3274 s	3271 s	νOH, νNH	polysaccharides, chitin, proteins
2925 m	2931 m	2932 m	2925 m	νCH_2_, νCH_3_ (aliphatic)	lipids, saccharides, proteins
2909 m	2908 m	2911 m	-	νCH_2_, νCH_3_ (aliphatic)	lipids, saccharides, proteins
2884 m	2874 m	2872 m	2872 m	νCH_2_, νCH_3_ (aliphatic)	lipids, saccharides, proteins
1636 m	1637 m	1637 m	1629 m	amide band I, νC=O	proteins, carboxylic acid
1370 m	1370 m	1372 m	1373 m	δCH_2_, δCH_3_, νCOO^−^	amino acid, carboxylic acid
1311 m	1309 m	1310 m	1315 m	δCH_2_, δCH_3_, νCOO^−^	amin oacid, carboxylic acid
1240 m	1240 m	1240 m	1251 m	νC-O, νP-O, νC-N (amide band II)	polysaccharides, phosphate esters, proteins
1149 m	1148 m	1148 m	1147 m	νC-O, νP-O, νC-N (amide band II)	polysaccharides, phosphate esters, proteins
1078 s	1079 s	1078 s	-	νC-O-C, νC-O	glycoside bridges in polysaccharides, β-glucans, chitin
1027 vs	1025 vs	1025 vs	1027 vs	νC-O-C, νC-O	glycoside bridges in polysaccharides, β-glucans, chitin
997 vs	997 vs	997 vs	993 vs	νC-O-C, νC-O	polysaccharides
911 m	930 m	932 m	-	_def_C-H_ring_	glucans, chitins, aromatic compounds
848 m	842 vw	847 vw	845 m	_def_C-H_ring_	glucans, chitins, aromatic compounds
803 m	803 m	803 m	801 m	_def_C-H_ring_	glucans, chitins, aromatic compounds
602 s	606 s	602 s	599 s	deformational, skeletal	mineral compounds
519 m	519 m	518 m	530 m	deformational, skeletal	mineral compounds

## Data Availability

The data presented in this study are available on request from the corresponding author.

## Supplementary Materials

The following supporting information can be downloaded at https://www.mdpi.com/article/10.3390/foods15132404/s1, Table S1: GC-MS analysis of ethyl acetate extract of *Pleurotus pulmonarius* samples; Table S2: GC-MS analysis of ethanolic extract of *Pleurotus pulmonarius* samples.
