# Supplementary material for: Substrate-Driven Modulation of Nutritional Composition and Bioactive Compound Profile in Pleurotus pulmonarius Cultivated on Diversified Agri-Waste
Source: Foods. 2026 Jul 7;15(13):2404. doi: 10.3390/foods15132404 (PMC13362493; doi:10.3390/foods15132404)
Supplement: Supplementary file 1 [file foods-15-02404-s001.zip › foods-4326888-supplementary.pdf]

Supplementary Materials

Table S1. GC-MS analysis of diethyl ether extract of *Pleurotus pulmonarius* samples.

Peak	RT	CAS#	Compound	PpS		PpC		PpP20		PpP100	
				Area	%TIC	Area	%TIC	Area	%TIC	Area	%TIC
1	10.49	33581-77-0	Glycolic acid, 2TMS	683256.9	0.02	2062418.94	0.11	1723110.66	0.08	1816543.56	0.10
2	10.66	7480-78-6	L-Valine, TMS	658516.12	0.02	-	-	-	-	-	-
3	12.68	55557-12-5	Laevulic acid, TMS	6347556.66	0.16	2369470.09	0.12	2671700.18	0.12	365492.69	0.02
4	13.4	55162-32-8	Hydracrylic acid, 2TMS	2080874.37	0.05	1640365.05	0.08	1894434.22	0.08	450388.18	0.02
5	14.15	55530-42-2	Isobutyric acid, 3-hydroxy, 2TMS	14893779.24	0.37	6197917.89	0.32	19358765.35	0.84	6879615.29	0.36
6	14.92	42411-50-7	Pantolactone, TMS	1120185.53	0.03	6417838.99	0.33	4078938.7	0.18	2701366.05	0.14
7	16.23	55124-90-8	3-Hydroxyisovaleric acid, 2TMS	17005104.01	0.42	1467462.08	0.08	1295773.48	0.06	880735.67	0.05
8	17.39	2078-12-8	Benzoic Acid, TMS	17961384.51	0.45	1413446.77	0.07	2110818.67	0.09	2335910.9	0.12
9	17.59	54890-08-3	2-Hydroxyisocaproic acid, 2TMS	7821223.24	0.19	152109.88	0.01	245970.6	0.01	-	-
10	17.67	18297-63-7	Urea, 2TMS	-	-	553958.24	0.03	897587.85	0.04	362636.03	0.02
11	18.43	55494-06-9	Octanoic acid, TMS	3115949.98	0.08	422178.93	0.02	526731.2	0.02	506245.06	0.03
12	18.74	5630-81-9	Ethanolamine, 3TMS	6109258.71	0.15	4444563.03	0.23	3399770.08	0.15	4687507.87	0.25
13	19.42	10497-05-9	Phosphoric acid, 3TMS	15969662.27	0.40	35041231.12	1.81	28754759.26	1.25	24988437	1.32
14	19.63	6787-10-6	Glycerol, 3TMS	143922540.8	3.58	44370359.48	2.29	44812152.9	1.95	41433560.96	2.19
15	19.74	6787-10-6	Benzeneacetic acid, TMS	6144924.38	0.15	503985.8	0.03	1270011.41	0.06	809587.19	0.04
16	20.92	40309-57-7	Butanedioic acid, 2TMS	8689387.78	0.22	18011861.71	0.93	15540121.68	0.68	5198743.69	0.27
17	21.46	55557-26-1	Methylsuccinic acid, 2TMS	1272079.12	0.03	1548622.52	0.08	1515416.53	0.07	1118669.26	0.06
18	22.02	10457-14-4	Uracil, 2TMS	1611563.58	0.04	989827.92	0.05	584118.68	0.03	649560.71	0.03
19	22.39	17962-03-7	Fumaric acid, 2TMS	3936544.51	0.10	1546608.46	0.08	1697707.28	0.07	248929.83	0.01
20	22.69	82326-11-2	Nonanoic acid, TMS	2952388.11	0.07	616736.92	0.03	1113148.23	0.05	878604.96	0.05
21	22.85	1012-12-0	4-Hydroxybenzaldehyde, TMS	437567.54	0.01	336773.74	0.02	224268.74	0.01	318290.06	0.02

23	24.67	-	Parabanic acid, 2TMS	5882701.41	0.15	2594589.44	0.13	1862453.13	0.08	992646.94	0.05
24	24.91	55494-07-0	Pentanedioic acid, 2TMS	2202482.68	0.05	2352047.27	0.12	4572963.78	0.20	1121563.01	0.06
25	25.35	55530-53-5	2-Methylglutaric acid, 2TMS	680242.37	0.02	818213.91	0.04	1428473.83	0.06	845578.23	0.04
26	25.67	-	L-Aspartic acid, 2TMS	692811.55	0.02	30713.2	<0.01	97350.33	<0.01	174286.85	0.01
27	25.78	55517-41-4	3-Methylglutaric acid, 2TMS	620429.05	0.02	1581925.43	0.08	1119982.39	0.05	1387835.61	0.07
28	26.85	55494-15-0	Decanoic acid, TMS	327859.59	0.01	82204.49	<0.01	206930.6	0.01	52769.48	<0.01
29	28.96	38166-11-9	Malic acid, 3TMS	24566041.09	0.61	11110675.1	0.57	10185659.93	0.44	6197250.05	0.33
30	29.15	74742-34-0	Pentonic acid, 2-deoxy-3,5-dihydroxy-, γ-lactone, 2TMS	1616481.04	0.04	2755963.8	0.14	2016680.19	0.09	976659.4	0.05
31	29.66	30274-77-2	L-5-Oxoproline, 2TMS	4740409.08	0.12	18783440.78	0.97	6852411.98	0.30	9671643.87	0.51
32	29.82	32381-52-5	Threitol, 4TMS	1890519.12	0.05	451476.56	0.02	256586.01	0.01	934652.52	0.05
33	30.17	2078-20-8	Cinnamic acid, TMS	7710413.61	0.19	1592188.71	0.08	1656889.55	0.07	1125126.96	0.06
34	30.36	55520-93-9	3-Methyladipic acid, 2TMS	3157751.59	0.08	7599259.44	0.39	3324384.42	0.15	2731699.35	0.14
35	32.23	27750-45-4	3-Phenyllactic acid, 2TMS	3146345.85	0.08	335488.51	0.02	274105.25	0.01	-	-
36	33.1	55530-58-0	Pimelic acid, 2TMS	2772413.17	0.07	632807.24	0.03	997910.58	0.04	631815.18	0.03
37	33.61	-	8-Hydroxyoctanoic acid, 2TMS	1589721.46	0.04	406929.09	0.02	586047.18	0.03	540140.02	0.03
38	33.72	2078-13-9	4-Hydroxybenzoic acid, 2TMS	15190941.84	0.38	1533600.36	0.08	1270349.69	0.06	888601.84	0.05
39	34.08	15985-07-6	Glutamic acid, 3TMS	3423398.42	0.09	422179.74	0.02	178623.21	0.01	545522.1	0.03
40	34.27	27750-57-8	4-Hydroxybenzeneacetic acid, 2TMS	4018639.64	0.10	531773.56	0.03	579865.97	0.03	131319.2	0.01
41	34.67	55520-95-1	Dodecanoic acid, TMS (Lauric acid)	2165726.12	0.05	1856402.81	0.10	2988184.08	0.13	1629732.35	0.09
42	36.68	43199-48-0	Octanedioic acid, 2TMS (Suberic acid)	2838765.78	0.07	871635.97	0.04	803961.74	0.04	587235.56	0.03
43	37.39	7449-14-1	Levoglucosan pyranose,3TMS	-	-	590257.2	0.03	834264.43	0.04	273966.1	0.01
44	38.84	6221-89-2	1-Tetradecanol, TMS	184916.05	<0.01	169093.24	0.01	400716.98	0.02	352909.13	0.02
45	39	2078-15-1	Vanillic Acid, 2TMS	33935.12	<0.01	612338.56	0.03	10345.04	<0.01	316475.11	0.02
46	40.26	17906-08-0	Azelaic acid, 2TMS	24423562.37	0.61	1558751.73	0.08	1865736.81	0.08	1309794.49	0.07
47	41.19	2347-40-2	Protocatechuic acid, 3TMS	139395.28	<0.01	445632.23	0.02	12817.26	<0.01	86317.07	<0.01
48	41.34	55556-70-2	Ornithine, N,N',N',O-4TMS	1061619	0.03	175124.36	0.01	-	-	251726.11	0.01

49	41.81	18603-17-3	Tetradecanoic acid, TMS (Myristic acid)	24991981.09	0.62	5461179	0.28	7832980.52	0.34	6526099.54	0.34
50	44.91	10517-30-3	p-Coumaric acid, 2TMS	2657586.7	0.07	202138.82	0.01	178298.7	0.01	203171.7	0.01
51	45.17	74367-22-9	Pentadecanoic acid, TMS	77358552.08	1.93	21894126.38	1.13	17290059.27	0.75	20168858.59	1.07
52	45.86	14317-07-8	D-Mannitol, 6TMS	26350545.14	0.66	35761889.14	1.85	15620904.92	0.68	44127961.04	2.33
53	47.31	-	Pantothenic acid 3TMS	604892.99	0.02	2461450.3	0.13	2070868.41	0.09	495383.85	0.03
55	47.66	82326-15-6	9-Hexadecenoic acid, TMS (Palmitoleic acid)	14862052.08	0.37	2938769.84	0.15	2645918.96	0.12	5020559.52	0.27
56	48.47	55520-89-3	Hexadecanoic acid, TMS (Palmitic Acid)	588318601.2	14.64	176108757.8	9.09	167284009.6	7.30	191409092.1	10.12
57	49.57	22396-19-6	Dodecanedioic acid, 2TMS	-	-	20761.9	<0.01	35058.49	<0.01	-	-
58	50.65	-	10-Heptadecenoic acid, (Z)-, TMS	5722941.68	0.14	1416684.04	0.07	1172561.01	0.05	1782517.01	0.09
59	50.77	2582-79-8	Myo-Inositol, 6TMS	2210544.39	0.06	2520854.77	0.13	708785.92	0.03	25823.11	<0.01
60	51.45	55517-58-3	Heptadecanoic acid, TMS (Margaric acid)	18730557.5	0.47	3056588.75	0.16	2706197.63	0.12	3419987.65	0.18
61	51.84	18748-98-6	1-Octadecanol, TMS	1706922.23	0.04	1757271.99	0.09	2734766.07	0.12	2758331.21	0.15
62	53.61	56259-07-5	Linoleic acid, TMS (9,12-Octadecadienoic acid (Z,Z)-,)	1312095125	32.65	428479449.8	22.11	484301764.8	21.13	495493415.6	26.19
63	53.74	96851-47-7	Oleic Acid, (Z)-, TMS	383184534	9.54	138448155.5	7.14	120291758.8	5.25	138096414.8	7.30
64	53.85	21556-26-3	Oleic Acid, (E)-, TMS	290400.77	0.01	4908230.67	0.25	6853914.73	0.30	7588014.62	0.40
65	54.44	18748-91-9	Stearic acid, TMS	244302470.5	6.08	41450643.67	2.14	31006579.85	1.35	47779600.59	2.53
66	54.7	55518-06-4	myo-Inositol, O-phosphate, 6TMS	6526083.88	0.16	2278987.93	0.12	4964832.04	0.22	2918401.05	0.15
67	56.16	-	9(E),11(E)- Conjugated Linoleic acid, TMS	9700292.55	0.24	5063319.69	0.26	5751855.43	0.25	6486020.01	0.34
68	59.09	-	11,14-Eicosadienoic acid, (Z)-, TMS	21862469.39	0.54	3084203.92	0.16	3875410.26	0.17	3614634.12	0.19
69	59.28	-	13-Eicosenoic acid, (Z)-, TMS	22485190.87	0.56	2503080.52	0.13	4135733.4	0.18	4682948.67	0.25
70	60.02	55530-70-6	Eicosanoic acid, TMS (Arachidic acid)	15889132.19	0.40	2326017.11	0.12	1928152.13	0.08	2292648.63	0.12
71	64.28	1188-74-5	1-Monopalmitin, 2TMS	8280149.2	0.21	19248113.82	0.99	25019291.33	1.09	23422081.14	1.24
72	65.16	74367-36-5	Behenic acid, TMS	20578586.34	0.51	2271710.89	0.12	1351578.19	0.06	1988778.37	0.11
73	65.82	53294-33-0	Adenosine, 4TMS	-	-	1807684.63	0.09	1906084.94	0.08	1711151.71	0.09
74	67.54	54284-46-7	2-linoleoylglycerol, 2TMS	12009974.58	0.30	13489631.93	0.70	22059397.37	0.96	19023789.38	1.01

75	67.61	56554-42-8	2-Oleoylglycerol, 2TMS	4677144.56	0.12	3234941.91	0.17	3901019.86	0.17	4696786.54	0.25
76	68.37	54284-45-6	1-Monolinolein, 2TMS	28285637.61	0.70	48785067.43	2.52	69102402.11	3.01	62799738.17	3.32
77	68.45	54284-47-8	1-Monoolein TMS	6999989.51	0.17	11507039.04	0.59	17937326.41	0.78	14401222.83	0.76
78	69.35	-	Trehalose, octa-TMS	180002942.2	4.48	78406115.26	4.05	71772402.71	3.13	87085644.33	4.60
79	69.61	111-02-4	Squalene	3640830.02	0.09	2348434.88	0.12	4307500.69	0.19	5118341.77	0.27
80	70	74367-37-6	Lignoceric acid, TMS derivative	68488582.32	1.70	6643949.18	0.34	5202062.06	0.23	6837407.87	0.36
81	72.39	-	NN (sterol)	9190854.97	0.23	8754276.6	0.45	10540767.32	0.46	10191773.04	0.54
82	73.5	-	NN	17337523.87	0.43	32429735.18	1.67	13858302.62	0.60	21351487.53	1.13
83	76.8	1856-05-9	Cholesterol, TMS	1531813.65	0.04	1164769.7	0.06	1812398.38	0.08	1791475.31	0.09
84	77.86	516-85-8	Dehydroergosterol, TMS	102712509.8	2.56	51988848.18	2.68	68759376.71	3.00	65836406.96	3.48
85	78.03	-	Ergocalciferol, TMS	5875876.18	0.15	4693150.11	0.24	9363352.74	0.41	945365.82	0.05
86	78.81	2625-45-8	Ergosterol, TMS	25979254.38	0.65	253346679.2	13.07	364308988.6	15.89	55298177.47	2.92
87	79.06	55527-93-0	Ergosta-7,22-dien-3β-ol, TMS	29843004.35	0.74	15255621.89	0.79	20298142.59	0.89	16685358.45	0.88
88	79.19	-	NN	39076562.33	0.97	6893572.56	0.36	20981641.33	0.92	14760736.9	0.78
89	79.96	-	Ergosta-5,7-dien-3β-ol, TMS	875756.68	0.02	11233487.61	0.58	24003529.54	1.05	18314543.08	0.97
90	80.27	18880-54-1	Ergosta-7-en-3β-ol, TMS	50732696.74	1.26	13962337.42	0.72	15824427.66	0.69	3736714.09	0.20
91	81.07	-	NN (sterol)	11027923.67	0.27	7994163.99	0.41	9611721.61	0.42	10056774.67	0.53
92	82.96	-	NN (sterol)	66981716.95	1.67	35620449.14	1.84	39623201.49	1.73	39582929.71	2.09
93	83.57	-	Ergosterol peroxide TMS	93121110.03	2.32	18431354.33	0.95	22828796.93	1.00	21233196.06	1.12
94	93.01	-	NN	63094423.93	1.57	9286995.33	0.48	15225946.38	0.66	9247160.53	0.49
95	95.31	-	NN (diglyceride of fatty acid)	5058293.02	0.13	97421943	5.03	177286438.1	7.73	119711141	6.33
96	96.39	-	NN (diglyceride of fatty acid)	6879488.89	0.17	111760725.9	5.77	195690580.1	8.54	142694114	7.54

Table S2. GC-MS analysis of ethanolic extract of *Pleurotus pulmonarius* samples.

Peak	RT	CAS#	Compound	PpS		PpC		PpP20		PpP100	
				Area	%TIC	Area	%TIC	Area	%TIC	Area	%TIC
1	10.48	33581-77-0	Glycolic acid, 2TMS	2138934	0.19	1889673	0.12	1769408	0.12	1489954	0.11
2	10.65	7480-78-6	L-Valine, TMS	3311196	0.29	952364	0.06	947729	0.06	449433	0.03
3	11.58	2899-44-7	Alanine, 2TMS	6677927	0.59	2753238	0.17	1544101	0.10	4899153	0.36
4	12.3	7364-42-3	Glycine, 2TMS	334656	0.03	269977	0.02	124079	0.01	56752	<0.01
5	13.06	18294-04-7	Oxalic acid, 2TMS	59443	0.01	279335	0.02	203642	0.01	103912	0.01
6	13.38	55162-32-8	Hydracrylic acid, 2TMS	350695	0.03	119428	0.01	159457	0.01	79558	0.01
7	13.59	-	L-Leucine, TMS	1369653	0.12	458251	0.03	393314	0.03	325060	0.02
8	14.13	55133-94-3	3-Hydroxyisobutyric acid, 2TMS	519120	0.05	117365	0.01	480347	0.03	357485	0.03
9	14.25	-	Proline, TMS	796089	0.07	297117	0.02	205045	0.01	161458	0.01
10	14.44	-	L-Isoleucine, TMS	726585	0.06	357806	0.02	329659	0.02	99613	0.01
11	16.21	55124-90-8	3-Hydroxyisovaleric acid, 2TMS	179754	0.02	-	-	-	-	-	-
12	16.55	7364-44-5	L-Valine, 2TMS	5169211	0.46	1141728	0.07	858492	0.06	1398891	0.10
13	17.84	18297-63-7	Urea, di-TMS	955904	0.09	3069976	0.19	932903	0.06	2985493	0.22
14	18.72	5630-81-9	Ethanolamine, 3TMS	3593652	0.32	4041450	0.25	5220162	0.35	4648221	0.35
15	19.16	15984-97-1	Leucine, 2TMS	5764604	0.51	334454	0.02	349146	0.02	952905	0.07
16	19.42	-	Phosphoric acid, 3TMS	49320977	4.39	95354093	5.88	70339646	4.74	50419547	3.74
17	19.62	6787-10-6	Glycerol, 3TMS	42329370	3.77	18175513	1.12	15008138	1.01	18762883	1.39
18	19.95	7364-47-8	L-Proline, 2TMS	3264146	0.29	171958	0.01	100108	0.01	343564	0.03
19	20.09	7483-92-3	Isoleucine, 2TMS	7894433	0.70	1497020	0.09	1158981	0.08	793194	0.06
20	20.48	5630-82-0	Glycine, 3TMS	2084670	0.19	1241554	0.08	1349570	0.09	1159577	0.09
21	20.9	40309-57-7	Butanedioic acid, 2TMS	7600043	0.68	2912610	0.18	3689205	0.25	1638909	0.12
22	22	10457-14-4	Uracil, 2TMS	227039	0.02	305071	0.02	126682	0.01	398631	0.03
23	22.09	38191-87-6	Glyceric acid, 3TMS	1007852	0.09	1093347	0.07	854373	0.06	698102	0.05

24	22.39	17962-03-7	2-Butenedioic acid, (E)-, 2TMS	18682523	1.66	2894893	0.18	6732917	0.45	753898	0.06
25	23.37	7364-48-9	L-Serine, 3TMS	13922659	1.24	2965168	0.18	2008884	0.14	2864913	0.21
26	23.7	55530-56-8	2,2-Dimethyl-3-hydroxybutanoic acid, 2TMS	1563783	0.14	-	-	-	-	-	-
27	24.55	64569-35-3	Threonine, 3TMS	9912712	0.88	1170813	0.07	201494	0.01	1405684	0.10
28	24.9	55494-07-0	Pentanedioic acid, 2TMS	802729	0.07	138926	0.01	331998	0.02	368428	0.03
29	25.65	-	L-Aspartic acid, 2TMS	1196647	0.11	6083304	0.37	7705621	0.52	4691892	0.35
30	26.56	55191-53-2	3,4-Dihydroxybutanoic acid, 3TMS	343928	0.03	240573	0.01	199630	0.01	155483	0.01
31	27.15	1177129-58-6	Homoserine, 3TMS	397986	0.04	-	-	-	-	-	-
32	27.95	-	Aminomalonic acid,3TMS	1492632	0.13	659876	0.04	535129	0.04	422818	0.03
33	28.98	38166-11-9	Malic acid, tri-TMS	95088795	8.47	83083396	5.12	96576761	6.51	51180528	3.80
34	29.39	55520-90-6	N-Isovalerylglycine, 2TMS	244228	0.02	-	-	-	-	-	-
35	29.66	30274-77-2	L-5-Oxoproline 2TMS (Pyroglutamic acid, 2TMS)	30187636	2.69	114384043	7.05	57626458	3.89	82438655	6.12
36	29.82	32381-52-5	Threitol, tetra-TMS	2549388	0.23	1217221	0.07	1301264	0.09	1142820	0.08
37	30.11	25258-02-0	Erythritol, 4TMS	6780766	0.60	-	-	-	-	-	-
38	30.19	39508-23-1	4-Aminobutanoic acid	15984179	1.42	17721453	1.09	20080540	1.35	20146913	1.50
39	31.52	38191-88-7	Erythronic acid, 4TMS	1123264	0.10	423627	0.03	374732	0.03	304361	0.02
40	32.39	55530-62-6	α-Hydroxyglutaric acid, 3TMS	5199455	0.46	185078	0.01	337134	0.02	52320	<0.01
41	33.32	55590-95-9	β-Hydroxy-β-methylglutaric acid, 3TMS	349075	0.03	-	-	-	-	-	-
42	33.79	2899-52-7	Phenylalanine, 2TMS	3028129	0.27	665345	0.04	1078673	0.07	1394809	0.10
43	34.07	15985-07-6	Glutamine, 3TMS	23085775	2.06	4109925	0.25	8756528	0.59	11510686	0.85
44	35.2	-	D-(+)-Ribono-1,4-lactone, 3TMS	3472734	0.31	3147653	0.19	3469714	0.23	3158374	0.23
45	37.71	66434-50-2	2-Aminoadipic acid, 3TMS	1509299	0.13	192306	0.01	128536	0.01	139683	0.01
46	38.06	32381-53-6	Ribitol, 5TMS	815833	0.07	755725	0.05	508944	0.03	742125	0.06
47	38.52	14199-72-5	Xylitol, 5TMS	5163162	0.46	2474315	0.15	2143778	0.14	1459836	0.11
48	38.65	25138-28-7	Arabinitol, 5TMS	829557	0.07	433778	0.03	361019	0.02	24254	<0.01
49	39.92	31038-11-6	α-Glycerophosphoric acid, 4TMS	2709151	0.24	18487410	1.14	10334895	0.70	6508768	0.48

50	39.98	53537-99-8	D-Fucitol, 5TMS	972375	0.09	-	-	-	-	-	-
51	40.18	57197-35-0	Ribonic acid, penta-TMS	15659985	1.39	3860501	0.24	10017249	0.68	1709315	0.13
52	40.55	30788-71-7	α-D-Methylfuranoside, 4TMS	2897047	0.26	371162	0.02	-	-	54235	<0.01
53	41.31	55556-70-2	Ornithine, 4TMS	324596	0.03	47911	<0.01	161991	0.01	1734296	0.13
54	41.54	-	α-Fructofuranose, 5TMS	1758450	0.16	562032	0.03	448787	0.03	77050	0.01
55	41.81	14330-97-3	Citric acid, 4TMS	24213624	2.16	21606111	1.33	42158155	2.84	14270431	1.06
56	43.12	7536-83-6	Tyrosine, 2TMS	6074004	0.54	2668655	0.16	1279360	0.09	1999879	0.15
57	44.12	55515-29-2	Gluconic acid, d-lactone, 4TMS	1713633	0.15	425544	0.03	365015	0.02	232823	0.02
58	44.46	3327-61-5	α-Glucopyranose, 5TMS	2178039	0.19	3542131	0.22	2577669	0.17	2041445	0.15
59	45.23	55528-75-1	Gulonic acid, γ-lactone, 4TMS	357007	0.03	550388	0.03	35206	<0.01	234215	0.02
60	45.35	51220-73-6	L-Tyrosine, 3TMS	594157	0.05	741628	0.05	768163	0.05	1395383	0.10
61	45.9	14317-07-8	D-Mannitol, 6TMS	156071345	13.90	399489607	24.61	321956668	21.72	404692262	30.05
62	46.11	14199-80-5	D-Glucitol, 6TMS	18505557	1.65	7344471	0.45	6315893	0.43	7129440	0.53
63	46.26	18919-39-6	D-Galactitol, 6TMS	4761447	0.42	3247138	0.20	4796342	0.32	2905840	0.22
64	46.58	29412-25-7	Chiro- Inositol, 5TMS	8473404	0.75	1048212	0.06	592857	0.04	322037	0.02
65	46.9	-	Cyclohexanepentol, 5TMS	699357	0.06	1696287	0.10	326143	0.02	304294	0.02
66	47.07	55530-80-8	D-Glucuronic acid, 4TMS	320269	0.03	118947	0.01	62973	<0.01	22116	<0.01
67	47.76	2775-90-8	β-Glucopyranose, 5TMS	2532565	0.23	6526248	0.40	6204730	0.42	3665092	0.27
68	48.19	34290-52-3	D-Gluconic acid, 6TMS	8885282	0.79	917632	0.06	913967	0.06	593313	0.04
69	48.36	55520-89-3	Palmitic acid, TMS	1533020	0.14	8521776	0.53	751932	0.05	691268	0.05
70	48.7	-	Sugar acid	2176941	0.19	707745	0.04	343177	0.02	281130	0.02
71	49.04	14251-18-4	Scyllo-Inositol, 6TMS	9630773	0.86	16107631	0.99	751896	0.05	479897	0.04
72	50.77	2582-79-8	Myo-Inositol, 6TMS	15359759	1.37	86848478	5.35	27981075	1.89	3563389	0.26
73	51.46	-	N-Acetyl-D-glucosamine, oxime, 5TMS	704395	0.06	163027	0.01	139511	0.01	105585	0.01
74	53.25	55429-34-0	Indole-3-butyric acid, 2TMS	10688	<0.01	-	-	-	-	-	-
75	53.38	56259-07-5	Linoleic acid TMS	3671388	0.33	2855539	0.18	2721695	0.18	2306668	0.17
76	53.55	21556-26-3	Oleic Acid, (Z)-, TMS	1155966	0.10	829877	0.05	443055	0.03	706360	0.05

77	54.38	18748-91-9	Stearic acid, TMS	324762	0.03	211617	0.01	187098	0.01	147449	0.01
78	54.68	55518-06-4	myo-Inositol, O-phosphate, 6TMS		<0.01	578029	0.04	531559	0.04	237120	0.02
79	55.22	-	NN (carbohydrate)	1345543	0.12	1646960	0.10	1568990	0.11	1249577	0.09
80	55.44	-	NN (carbohydrate)	1700059	0.15	1882049	0.12	1920667	0.13	1120345	0.08
81	57.68	55520-79-1	Glucose-1-phosphate, 6TMS		<0.01	3894472	0.24	2233295	0.15	1356489	0.10
82	58.99	33800-37-2	Mannitol phosphate, 5TMS	571262	0.05	7843198	0.48	4246512	0.29	2481630	0.18
83	60.53	10457-16-6	Uridine, 3TMS	1989089	0.18	5514407	0.34	2757965	0.19	3988516	0.30
84	66.1	-	D-(+)-Turanose, 8TMS	2735363	0.24	5500018	0.34	2252504	0.15	2039799	0.15
85	66.87	19159-25-2	Sucrose, 8TMS	426688	0.04	478947	0.03	504716	0.03	391573	0.03
86	69.4	-	Trehalose, 8TMS	426200940	37.95	619267138	38.16	705283404	47.58	597602437	44.37
87	70.91	-	Cellobiose, 8TMS	185048	0.02	165253	0.01	48712	<0.01	25391	<0.01
88	71.52	-	Maltitol, 9TMS	67691	0.01	40616	<0.01	18433	<0.01	17284	<0.01
89	72.18	-	Maltose, oxime, 9TMS	4257156	0.38	6880226	0.42	1837296	0.12	1534313	0.11
